# The Relational Vulnerability of People Experiencing Multiple Exclusion Homelessness (MEH) in Spain

**DOI:** 10.3390/ijerph181910275

**Published:** 2021-09-29

**Authors:** Hugo Valenzuela-Garcia, José Luis Molina, Miranda J. Lubbers, Jorge Grau

**Affiliations:** 1 CREIS, Department of Social Anthropology, Universitat Autònoma de Barcelona, 08193 Bellaterra, Spain; Hugo.Valenzuela@uab.cat; 2 GRAFO, Department of Social Anthropology, Universitat Autònoma de Barcelona, 08193 Bellaterra, Spain; MirandaJessica.Lubbers@uab.cat (M.J.L.); Jordi.Grau@uab.cat (J.G.)

**Keywords:** homelessness, relational vulnerability, charities, social network analysis, exclusion, Spain

## Abstract

This paper draws on research analyzing the emotional and relational impacts of poverty and exclusion on charities’ clients in Spain since the 2008–2009 economic crisis, including people experiencing multiple exclusion homelessness (MEH). The study adopts a mixed-methods approach in which twenty in-depth cases were collected in different geographical locations, including twelve cases experiencing MEH. Unlike other disadvantaged groups, those affected by MEH suffer material shortages, traumatic experiences, psychological disorders, physical illnesses, and a high degree of relational vulnerability, as reflected in the structure and composition of their personal networks, which tend to be smaller in size, with just a few weak and temporary contacts, and with care professionals playing an important role. These charity users often need long-term socio-sanitary care, which challenges public and private health-care systems. Therefore, our contribution to this Special Issue is directed toward improving understanding of the relational characteristics of severely excluded people, how social support affects their personal networks, and the challenges this assistance poses to care services.

## 1. Introduction

During the last decade, Spain has suffered from the widespread effects of downward mobility, which has revealed that poverty can reach social strata that have been relatively protected from social exclusion in the past (e.g., the middle classes; see [1]) but have now become more disadvantaged and find themselves in a situation of despair. Although traditionally Mediterranean societies such as the Spanish depended heavily on the family to provide material, social, and emotional resources, the lasting effects of the 2008–2009 financial crisis and the withdrawal of public aid have deeply eroded this source of informal social support. In many cases, the family can no longer help because there is no one to provide help [2,3]. For this reason, a growing number of individuals are turning to charities. In 2019, Humancare (a pseudonym), one of the main charities in Spain, dealt with 1,403,269 people and invested nearly 337 million euros (of which 71% were private donations) to help out those without resources. During this period, 72.7% of requests for aid were directed to the reception service, which was an alarming increase in new users. While in 2019, Humancare managed just 5000 places for the homeless persons and dealt with between 35,000 and 38,000 people, the Coronavirus COVID-19 pandemic has increased the need for help by 57%. The number of aid requests has skyrocketed to 40,000, with over 20,000 homeless people having to be taken in by social shelters.

### 1.1. Post-2008 and COVID-19: It Never Rains but It Pours

Spain is still suffering the devastating consequences of the 2008 financial crisis. From 2009 onwards, unemployment peaked at 30%, with a high percentage of long-term jobless without any social benefits, and Spain registered the second highest severe poverty rate in the European Union (6.9%), behind Romania [4]. In 2020, before the pandemic spread, 18.4% of the population (8.5 million Spaniards) was already in a situation of social exclusion, 4.1 million were in a condition of severe social exclusion and, of them, 1.8 million (compared to 600,000 in 2007) required urgent intervention to guarantee their basic subsistence. Between 2011 and 2013, when help was most needed, the government started to introduce severe austerity measures to reduce the deficit and pay off sovereign debt, implying a budget reduction of 45% in public health and 13.3% in social services. As a spokesman for the Association of Social Services pointed out, social services suffered a cut of 2.200 million euros (a fall of 13.3%) on a budget of 14.982 million euros in 2013, meaning “a miserable and unjustifiable abandonment of citizens in situations of greater vulnerability” [5].

These negative socioeconomic figures have fueled the risk of social exclusion (The European Council defines the *population at risk of poverty* as people living in households that meet at least one of the following three conditions: (a) having income per capita, on an equivalence scale and after transfers, of less than 60% of the median national income (the value of the poverty threshold in Spain was 6279.7€ in 2018); having all household members under 59 working below 20% of their potential hours; and, third, having at least four of the following conditions of material deprivation: (i) being unable to pay the mortgage or rental costs or utility bills; (ii) being unable to heat one’s home; being unable to meet unexpected expenditure; (iii) being unable to eat meat or protein regularly; (iv) being unable to afford a holiday; (v) being unable to afford a television; (vi) being unable to afford a washing machine; (vii) being unable to afford a car; (viii) and being unable to afford a telephone. *Social exclusion* is one of the consequences of sustained poverty, implying the lack of resources, rights, goods and services, and the inability to participate in regular activities, whether in economic, social, cultural, or political arenas. Finally, *severe or extreme social exclusion*—as in homelessness—implies a higher degree of disenfranchisement, social discrimination, and the accumulation of difficulties (severe health problems, substance abuse, prostitution, etc.) that stigmatize the individual and hinder their normalized participation in a community). We talk about exclusion and not just poverty because exclusion implies the existence of structural, dynamic, and multidimensional factors (i.e., lack of economic resources, employment, or social services) that seriously hamper access to educational and health resources [6] (p. 34). For this reason, within the European Union framework, from the 1980s onwards, the term “social exclusion” has progressively replaced “poverty” in order to shift to a multidimensional view of poverty that, beyond material resources, considers the intensity and form of individuals’ social ties alongside the characteristic of social protection systems [7]. This new approach pays more attention to “relational vulnerability”, i.e., the weakening or absence of community links, social support, and social capital [3,8,9].

Regarding the concept of relational vulnerability, the literature on social support has demonstrated the importance of personal relations for a variety of outcomes related to well-being, resilience, and health [10] by providing resources either directly (including material and emotional support) or indirectly to people who can be reached in case of need. Some authors have reviewed the use of personal network analysis for measuring access to or the availability of social support through name generators [11,12]. In this vein, Lubbers et al. conclude that the support networks of the poor tend to be smaller than those who are not poor [13], homophilic (i.e., relationships tend to occur between individuals who share a similar socioeconomic status) [14], and temporary (since they can only provide aid for a limited period) [15]. We do expect that the personal networks of those affected by MEH would be still smaller [16], yet most homeless people still have sporadic contact with their families.

Although the conceptual leap from poverty to exclusion was welcomed by social scientists, in recent decades, social policies have more often been devoted to emergency programs than used to address structural inequalities, leaving aside the growing groups of the “working poor” (i.e., people that fall under the official definition of poverty despite having a job) and the “homeless” [17] (p. 585). Although the working poor, a relatively new phenomenon, present understandable research gaps, the little data available about the homeless are striking, since it is a group that is particularly vulnerable to cumulative disadvantage and relational dissolution [18]. As Minnery and Greenhalgh point out: “Rather than being linked to poverty or deviance, homelessness is increasingly being viewed as a component or expression of social exclusion […] a process by which individuals and groups become isolated from major societal mechanisms providing social resources” [19] (p. 645).

### 1.2. Homelessness: General Traits and Relational Vulnerability

Apart from some studies in the United States, longitudinal research on large samples of the homeless is virtually unknown, and little research has been conducted on their living conditions and behavior [20] (p. 467) [10] (p. 583), daily sociability [17,21], or the way socioeconomic assistance impacts on the social networks of people experiencing multiple exclusion homelessness (MEH). It is revealing that in Spain, the National Strategy for the Homeless, launched in 2015, was abandoned in 2020 without being implemented due to “a lack of budget” [22,23] and the fact that the National Institute of Statistics (INE) has had no reliable data on the number of people living on the streets since 2012. Nevertheless, based on municipalities’ calculations, it is estimated that more than 33,000 people, with a notable increase in youngsters and people over 65 years old, are homeless in Spain. In 2019, the City Council of Barcelona counted 3700 people in this situation, while Madrid estimated that there were 2700 homeless people, of whom 650 slept on the street every day (see Sindicato Asambleario de Sanidad, SAS, 13 January 2021).

Mainstream research in Europe, the US, and Japan highlights some common traits in homelessness, despite the differences in countries, welfare systems, and policies. In the US, homeless single adults are predominantly male (70–80%) with a history of alcohol and/or drug abuse and/or dependence (60–80%), with a high percentage (about 20–40%) of physical (mainly respiratory problems and hepatitis) and mental illness (commonly severe depression and, to a lesser extent, schizophrenia) [20] (p. 461). Similar data are found in Japan, although the phenomenon of homelessness is relatively ‘new’ [24]. In a systematic review of the literature (from 1970 to 2007) in Europe, Philippot et al. [25] remark that most of those affected with MEH are unemployed, live alone (95% unmarried), remain in the homeless state for many years and, as a common psychological risk factor, were separated from their families at an early age. These data are congruent with the findings of Muñoz and Vázquez [26] in Spain, who more than two decades ago had already calculated a rate of 70 homeless individuals per 10,000 inhabitants each year, which is a percentage that has clearly increased today.

Among the MEH-affected group, the high number of suicide attempts and solitary deaths prove their vulnerability and the destructive outcomes of severe exclusion. According to Subirana, every six days, a homeless person dies in Spain, and living on the streets shortens life by between twenty and thirty years [27]. Rough sleeping, violence, stress, poor diet, alcohol or drug use, illness, and limited access to health care (less than 50% have the card needed to access the public health system) explain such high rates of mortality. In Barcelona, according to the Grassroots organization Arrels, 64% of homeless people were attended by emergencies in only six months, and 31.6% suffered physical or verbal aggression. In the case of women, this percentage rises to 40.6% [27]. As Desjarlais [28] (p. 122) shows in his ethnography of Boston’s homeless, “dwelling on the street could mean months of living on the margins of language, communication, and sociability […] A person’s very nature changed, particularly one’s capacity from communicating with others. The longer people lived on the streets, the less they lived as social beings”.

### 1.3. MEH and the Charities

As we have observed, most people who resort to charities suffer from an urgent material or informational need, such as paying a bill, buying food and clothes, or seeking legal advice [3]. By definition, urgent assistance rarely favors long-term relationships, whether with the institutions, their workers, or other users, nor does it transform the social network of the individual being assisted. Conversely, those subject to MEH require long-term care, providing them with more possibilities to create and enhance social relationships. At the same time, poor access to assistance is a significant concern for such individuals because of organizational and bureaucratic barriers and because they often feel stigmatized and treated badly when they seek health care [16]. In general, when people knock on a charity’s door, it is because there are not many more alternatives, either because their networks cannot provide resources or because they are reluctant to ask for further help [29]. This situation is far more complex in the case of MEH, who are exposed to a profound sense of social erosion:

The longer the homelessness period, the higher the prevalence to display networks composed of riskier members when compared to men intermittently homeless during that time and the greater social network fragmentation. While intermittent homelessness affects network composition in ways that may be addressable with existing interventions, chronic homelessness fragments networks, which may be more difficult to address with those interventions. These findings have implications for access to social support from network members which, in turn, impacts the resources homeless men require from other sources such as the government or NGOs [21] (p. 112).

These long-term spaces of care (e.g., soup kitchens or social shelters) are often niches that are protected from the rest of our modern and competitive society and that offer an opportunity to socialize and establish relationships without the emotional impact of long-term and emotionally dense relationships [30] (p. ii), [31] (p. 12), [32] (pp. 3–4). Although specific social dynamics have been observed between users and professionals in these spaces [17,33], as well as specific forms of cooperation and reciprocity among users [34], further research is needed in this direction.

In this article, we measure relational vulnerability by examining personal networks. Specifically, this paper addresses the following questions: How does MEH affect the support networks of MEH-affected people? What challenges does their long-term care pose to charities? To answer these questions, we use a mixed-methods approach combining ethnography with personal network analysis. Our contribution will provide better knowledge of the relational vulnerability of MEH-affected people and the socio-emotional nature of long-term attention in the charity field.

## 2. Materials and Methods

This research, carried out between 2019 and 2020, collected twenty cases in four charity centers distributed throughout Spain (Castelló, Madrid, Albacete, and Catalonia), as well as contextual data and numerous testimonies from volunteers and social workers (in-depth interviews with six technicians and four volunteers).

### 2.1. Mixed-Methods Approach

The research adopted a mixed-methods approach drawing on primary sociodemographic data, ethnography, and two kinds of interviews: first, semi-structured interviews aimed to collect sociodemographic data, life histories, and introspective insights about the experience of poverty and exclusion; and second, computer-assisted personal interviews (CAPI) were used to obtain egocentric or personal network data, thus complementing the qualitative methods with structured network data [35].

The ethnographic perspective allowed us to document the interactions between the various actors involved in the social fabric (users, volunteers, social workers) and to analyze the context of social interactions (social shelters, soup kitchens, medical centers, etc.) through multi-sited fieldwork [36]. The semi-structured interview, which usually took around two hours, consisted of three parts. In the first part, individual data (place of birth, age, marital status, number of children, level of studies, employment situation, informal income, incoming social benefits, etc.) were asked for. The second part explored the individual’s life history and his/her economic, labor, health, and family situation. Finally, the third part explored the subjective and emotional aspects of the experience of poverty. The interviews were recorded and transcribed with the explicit permission of the interviewees and later analyzed through coding and contextual analysis.

For the personal network analysis, we used Egonet, which is a software package that integrates questionnaires, analysis, and the visualization of personal networks. The approach consists of analyzing the social relationships that an individual (i.e., ego) maintains with the other network members, i.e., his or her alters. We used several name generators to capture alters who provided different types of support (material, emotional, etc.). Furthermore, we collected data on the characteristics of the alters themselves. For the purposes of our study, we selected the following characteristics: sex, type of relationship with ego (father, mother, friend, professional worker, etc.), perceived degree of proximity (I feel close, very close…, etc.), interaction frequency (I meet/communicate very often, often, rarely, etc.) and duration (less than 1 year, between 1 and 5 years, more than 5 years, a lifetime), perceived financial situation from ego’s point of view (better than me, like me, worse than me) and occupational background (high, medium, or low professional skills). Finally, to visualize each network’s structure, we gathered information on the relationship among the alters (“Does Y1 know Y2, Y3, …, Yn?”). Thus, the analysis of personal networks makes it possible to analyze empirically the relational dimension of poverty through the characteristics of the users’ networks and the volume and type of support that flows through them.

The phase of preparing, collecting, processing, and storing personal data follows basic rules, such as anonymizing the data or removing names or other direct identifiers. The names used in this paper are pseudonyms, including that of the charity, and we have removed all sensitive information. All participants were adequately informed of the study’s objectives and procedures, of the voluntary nature of their participation in the study, and of their right to end the interview at any time. The researcher also requested the interviewee’s explicit consent to the interview being recorded before the interview started.

### 2.2. Sampling

The sample was intentional, aiming at maximizing the diversity of cases and situations in order to acquire a broad vision of the phenomenon. In the present paper, we focus specifically on the cases of users who have suffered severe exclusion and homelessness (i.e., MEH): we found twelve individuals with this profile, nine men and three women, who displayed certain regularities in their personal networks, exclusion processes, and sociodemographic factors. The mean age of informants was 52.2 years in a range from 41 to 65 years (and a median of 51 years). Significantly, in this subsample, all the individuals were single, divorced, or had no known stable partner, except for three individuals who had a temporary relationship with other users of the charitable institution (a couple from the sample and a man with another user). Regarding origins, eleven were born in Spain and one was born abroad. At the time of the interviews, only three were working, the remainder counting as long-term unemployed, although in the past, most of them had had different kinds of temporary employment, e.g., as a cook, artist, factory worker, or hairdresser). Eight out of the twelve had only a basic educational background; two had achieved intermediate educational levels (baccalaureate or professional training), while the remaining two lacked any academic background. In the past, they had all had some problems with substance abuse in the form of alcohol or drugs, and a couple of them were still dependent on it. They were all suffering from different health problems, particularly psychological disorders. At some point in their lives, most had lived on the street for varying lengths of time, from a few months to several years. At the time of the interview, most were renting a room, had been given accommodation by a relative, or were staying in a social shelter under the charity’s supervision.

## 3. Results

This section presents the life backgrounds, support networks, and impact of charities on users’ personal networks. We first describe Laureano’s case, which exemplifies those who have to resort to charity sporadically because of episodic financial shortages. Due to their relatively wide social links, they usually manage to overcome the impasse despite their financial vulnerability. Next, we will present the specific cases of people experiencing MEH whom the charitable institution deals with at different points in the caring process.

### 3.1. Temporary Need and Specific Assistance for Poor People

From 2008 onwards, Humancare experienced a dramatic increase in assistance for people needing specific material resources, such as food, clothing, and the timely payment of bills, or information support regarding job training, legal aid for mortgage payments, evictions, etc. New users generally go through a period of socioeconomic vulnerability caused by unemployment, the accumulation of debts, and a temporary lack of economic resources. In these cases, contact with the institution is limited to a specific aid provision that prevents claimants from falling into a more difficult situation of exclusion, but the institution’s casual contacts rarely establish lasting social ties. In most cases, even though their close contacts (family and friends) can barely provide them with enough material resources, they still retain a broad social network.

Laureano’s case provides a good example of this sort of charity client. In 2012, at 62 years old, he was diagnosed with several diseases (fibromyalgia, chronic fatigue, and Crohn’s syndrome). After a brief period of medical leave on top of 25 years working in the IT sector, he was fired. The flexible labor laws enforced by the right-wing government during nearly a decade in power substantially reduced the costs of layoffs. Accordingly, Laureano received a tiny, insufficient pension. As the sole breadwinner in a family also including a wife and two teenage children, the debts began to accumulate. Initially, he asked family members for help. After a while, he felt ashamed and turned to charities instead, which provided him with food and covered some overdue bills.

**Figure 1:**
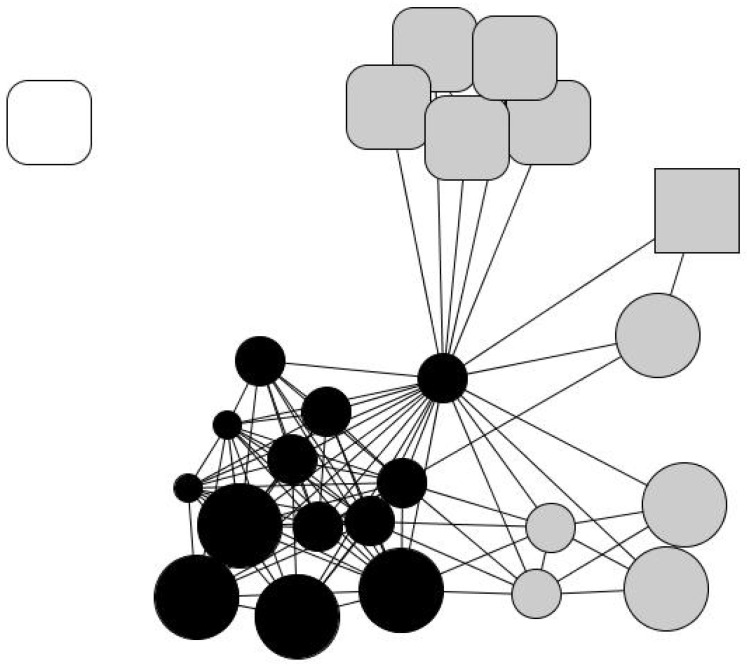
Laureano’s personal network. Node color indicates the type of relationship: family members (black), friends and acquaintances (gray), and professionals (white). Node size indicates ego’s perception of alter’s status: big (ego perceives that “alter has a better socioeconomic situation than me”), average (ego perceives that “alter has a similar situation as me”) and small (ego perceives that “alter has a worse socioeconomic situation than me”). Node shape indicates the length of the relationship: a triangle (less than 1 year of relationship), a square (between 1 and 5 years of relationship), a rounded rectangle (more than 5 years), and a circle (lifetime).

Laureano’s personal network is relatively large, at 25 members: 44% are friends and over 50% are family members. He maintains regular contact with 60% of his old friends and feels “very close” to 88% of his contacts. He believes that 52% of his contacts enjoy a better socioeconomic situation than himself and that the same percentage have had above-average educations. In his network, his wife and children are very central, as they connect different social spheres, namely relatives from both sides of the family and friends from different contexts. His family and close friends may not provide substantial material resources, but they help him with minor services, favors, and gifts, as expressed in the qualitative data. Although he has had to cut down on consumption expenses, he is not living in conditions of severe exclusion. His subsistence and vital necessities, such as medical expenses, his mortgage, and basic material needs, are covered. His personal network has a significant number of strong ties consisting of long-term relationships in different social contexts and with a comfortable socioeconomic status that provides him with socio-emotional support. In other words, his personal network retains some features of the typical Spanish middle-class person. His contact with the charity is depicted in his drawing of his personal network (Figure 1) with Humancare’s social worker placed in the upper left corner, completely disconnected from any of Laureano’s other contacts.

### 3.2. Cases of Persons Experiencing MEH

**Figure 2:**
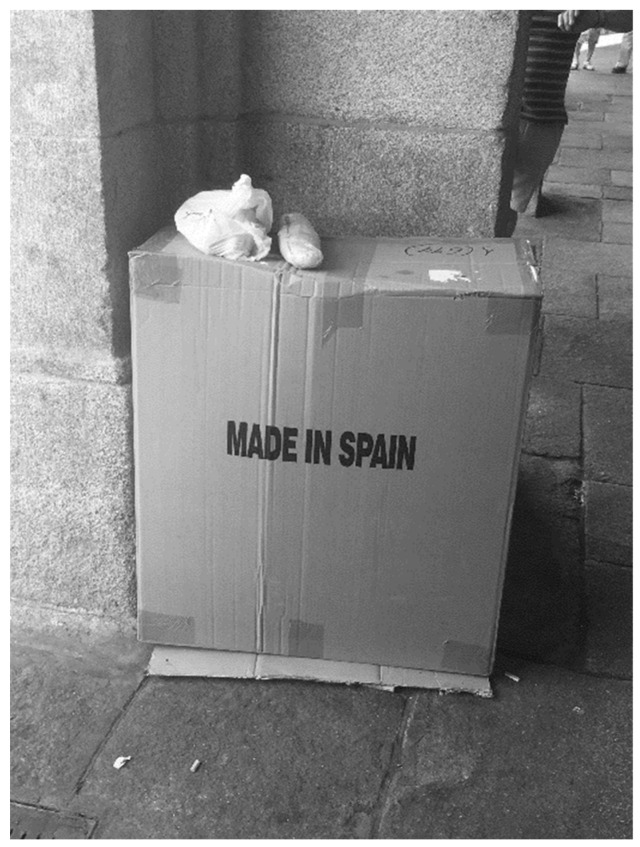
A makeshift shelter in Madrid, Spain.

People subject to MEH present several associated problems beyond economic want (Figure 2). In comparison with Laureano’s case, their personal networks are smaller, and they have little social capital.

Dora, for instance, is a 47-year-old Brazilian transgender person. Some years ago, she arrived in Spain, fleeing the world of prostitution and drugs in her natal *favela*. In Spain, she married a man who mistreated her, and in running away from him, she ended up on the street without any other social support. She slept in ATM lobbies and collected leftovers from restaurants. Then, she started to “hear voices” inside her head caused by incipient schizophrenia, which prompted her to try and take her own life on several occasions. Once, she swallowed sodium hydroxide, burning her larynx and tongue. She suffered multiple forms of stigma and abuse on the street against her ethnic origin, her gender identity, her economic condition, and her mental distress. On one occasion, someone tried to burn her alive at night, although she managed to get away.

When Dora went to the charity, she was physically impaired, lacking mobility and suffering from atrophy from sleeping on the floor, as well as presenting with memory loss and a lack of communication and social skills alongside severe material needs. She also lost her only suitcase with her phone containing all her contacts.

As in other cases we observed, Dora alternated life on the street with periods in shelters in different cities (staying in shelters is limited to a few weeks). Her nomadism made it challenging to carry out a proper medical diagnosis and follow-up. She was only diagnosed with schizophrenia after the police rescued her from the street after an anonymous call reported her suffering from confusion and hallucinations. Later, she was referred to a psychiatric hospital, where she stayed for several years until she left and ended up at Humancare. Eventually, the charity offered her a single social apartment.

Mutatis mutandis, Dora’s case bears close similarities with those of Kike, Gregor, Inma, and Monroy. They all had small and unstructured social networks and were expelled or had run away from their homes. “(My family) threw me out with my clothes on the street, like shit (…) my children don’t even look at my face”, says Gregor. In these cases, the relationship with family members has deteriorated so much that, as one worker put it: “When we get in touch with their family members, they usually hung up on us or sent us to hell. They don’t want to know anything. At that time that person has no family, he has burned them, there is no turning back, and they must recognize it”.

Nevertheless, although informants’ contacts with their families could be irregular and conflictual, some contact with close friends and family by telephone or via social media platforms such as Facebook was not entirely unusual. Thus, Dora, Inma, and Gregor telephoned their families from time to time. Gregor resumed contact with some of his brothers when their mother fell sick. Monroy called his mother once a week and had engaged in Facebook exchanges with old friends. Inma and Sara stayed in contact with their children, who had been adopted or were in foster homes.

All of them also suffered from severe psychological distress. The frequency of confessed suicide attempts found in the sample is alarming: “My life has no meaning. I’d rather get out of the way”, said Inma, a woman in her forties paired with Gregor. Similar feelings were expressed by Kike, Sara (a woman also in her forties who begged on the street), Monroy and, as already mentioned, Dora.

These informants were all exposed to stigma and structural violence as well. While we were interviewing Monroy on the terrace of a bar, the owner suddenly appeared shouting: “I don’t want this kind of people in my bar!”, and we had to leave. Dora told us that she was not even allowed to look at the women’s clothing through shop windows from the outside because, according to the sellers, she “scared off the clientele”. She missed just “having a coffee on a terrace while chatting with someone”, which is an indication of how isolated she felt. The same situations were reported by Kike, who was often asked to leave public spaces when living on the street, and Eduardo, who, when entering a supermarket, was often followed by shop assistants for fear he would steal alcoholic drinks. According to Sara, homelessness means that “You live in a different world. You are out of society.”

### 3.3. MEH and Long-Term Attention

The transition from the street to the care institution involves a fragile process. In all cases, individuals showed great distrust when resorting to charity aid, and even after doing so, they showed resistance to being treated, which was probably caused by the addiction and pathologies and by the sustained structural violence they had suffered during their lives. As one social worker observed:

The lives of these people are disjointed, and the soup kitchen is a place where many go, eat, and leave. At the beginning, the objective of the care is rather than rescue the person to undertake work to restore basic patterns and routines.

Severely excluded users usually require prolonged and in-depth social and health care, but in the initial stages, there is a high risk of recidivism (particularly in the case of addictions) because their personal networks become even weaker. On the one hand, they must move away from the harmful situations of the past, while on the other hand, they are encouraged to create new relationships in *healthy* environments (free from temptations in case of previous addictions).

Gabino, 57 years old, consumed heroin for more than fifteen years, although he tried to hide it until his whole social world collapsed: he was fired, broke with his family and friends, and his partner left him. After some time on the streets, he turned to the Addictive Behaviour Unit, which is a service provided by Humancare. When we interviewed him, he was “clean” (he had overcome his addiction more than a decade ago) and had started a part-time job in a public library. However, he had not managed to mend his social ties: stigma and a fear of rejection prevented him from talking about his life openly, so he avoided close relationships with his new co-workers. His parents had passed away, and the only stable relationships he maintained were with a friend, a social worker, and his younger brother. So, even years later, his network was still minimal and fragile.

Kike’s case has some close similarities with Gabino’s, although he explains his experience as a “success”. Raised in a large working-class family, he started to consume heroin, committing minor crimes to sustain his addiction. Over a decade, he broke with his family, lived on the streets, went to prison several times, was infected by HIV, and made several suicide attempts. By chance, he met an altruistic lawyer committed to prisoners’ reintegration who put him in touch with a small Jesuit congregation that took care of him and directed him toward Humancare. At the charity, he went through rehabilitation and found crucial emotional support. Later, the charity offered him a job as a receptionist, and he started to recover his contacts with family members and made new close contacts with social workers, professionals, and therapists. Kike, today fifty years old, overcame his addiction 25 years ago, but HIV had caused a deterioration in his physical appearance, and he cannot avoid stigma: “Facing society is more difficult than giving up drugs,” he says. He lives with his younger brother, and today, his personal network is broad and integrated, but mainly “institutional”.

**Figure 3:**
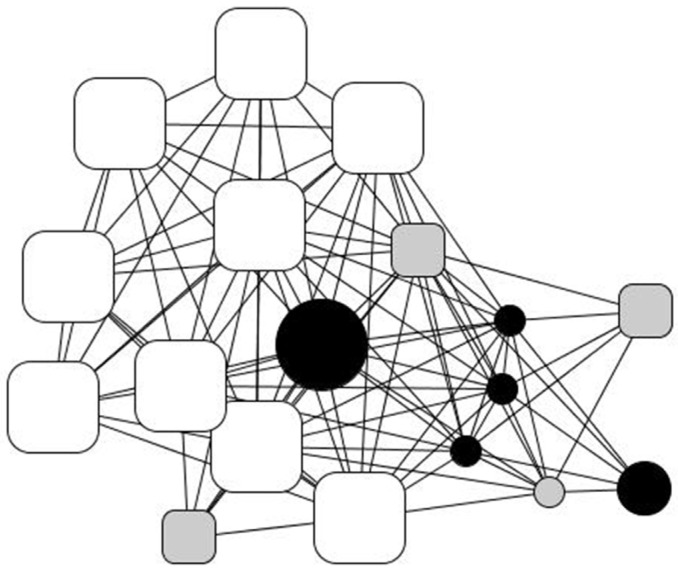
Kike’s personal network. Node color indicates the type of relationship: family members (black), friends and acquaintances (gray), and professionals (white). Node size indicates ego’s perception of alter’s status: big size (ego perceives that “alter has a better socioeconomic situation than me”); average size (ego perceives that “alter has a similar situation as me”), and small size (ego perceives that “alter has a worse socioeconomic situation than me”). Node shape indicates the duration of the relationship: triangle (less than 1 year of relationship), square (between 1 and 5 years of relationship), rounded rectangle (more than 5 years) and circle (lifetime).

His personal network (Figure 3) consists of eighteen people to whom he feels “very close”. Roughly half of them are strong ties: family members (22%), with the notable centrality of his older brother, who supports him in several ways, his younger brother, his sister, and his two nieces, who in turn often resort to him, as well as old friends from humble backgrounds (25%). The rest comprise professionals from the social institution that Kike refers to as “my other family”: a cohesive and well-to-do group of people who provide high social and emotional support. Therefore, his network is broad and cohesive, very heterogeneous, and offers Kike chances to reciprocate (e.g., they constantly ask him for small and material help, involvement, advice, support, and company) that, as he puts it, “helped me to recover my self-esteem”.

Julio and Andrés, both in their fifties and long-term Humancare users, share a social apartment in Castelló, where they are starting to reconstitute their lives from the ruptures left by long periods of alcohol addiction. In the past, they exhausted all their financial resources, unsuccessfully stayed in several detoxification centers, and broke off relationships with their relatives and friends: “I’ve lost twenty years of my life, from twenty to forty,” says Julio.

**Figure 4:**
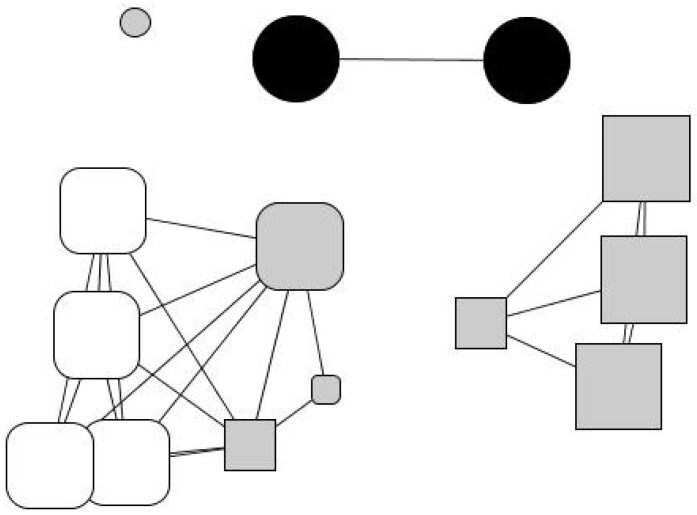
Julio’s personal network. Node color indicates type of relationship: family members (black), friends and acquaintances (gray), and professionals (white). Node size indicates ego’s perception of alter’s status: big size (ego perceives that “alter has a better socioeconomic situation than me”), average size (ego perceives that “alter has a similar situation as me”) and small size (ego perceives that “alter has a worse socioeconomic situation than me”). Node shape indicates time of relationship: triangle (less than 1 year of relationship), square (between 1 and 5 years of relationship), rounded rectangle (more than 5 years) and circle (lifetime).

Julio went through a period living on the streets until he accessed a social shelter. He particularly valued the supervision it provided him with, which helped him establish routines and habits, prioritize needs, and have some control over savings. He also appreciated the material aid: the charity paid some of his debts, provided him with a temporary job, and covered his medical treatment. The charity also assigned him a social worker, Mariluz, who established an emotionally intense and dependent relationship with him. As Julio explains, this relationship has acquired a maternal dimension: “I respect Mariluz like a mother. What I like the most is her confidence. We are not friends, we are something else… And I’m going to tell you what we are: a family”. In his personal network (Figure 4), now considerably wide, fourteen people are distributed into four subgroups: an isolated friend (in the upper-left corner), two distant relatives (his aunt and cousin, upper side), a group of new friends (right-hand margin), and a group of users and professionals from institutional contexts (left-hand side). In his network, 79% of his contacts are recent (last five years), indicating the creation of a new social fabric away from the context of addiction. However, the isolated friend is someone Julio wants to avoid because he considers he is a bad influence, and the new groups of friends are a group of young people who work in a gambling house and share high consumption habits.

Julio perceives 71% of his network members as having a “better socioeconomic situation” than his (the professionals, distant relatives, and new friends), while 14% have a “similar situation” and 14% are socioeconomically worse off. According to Julio, most of his contacts (except for 28% of the institution’s professionals) have received only a little (40%) formal education, if any (28%).

Andrés, Julio’s flatmate, started working as a bartender at an early age. His father’s sudden death triggered a depression, so he began to drink. Things went awry when he lost his job and stopped paying his mortgage until he was evicted. Some old friends persuaded him to visit the Addictive Behavior Unit (UCA). From there, after some months of treatment, he lived for a while in a social shelter until he was transferred to a small shared social house and finally settled in the social apartment he shares with Julio. During these last months, he has gone on training courses and worked in a clothing recycling company depending on the charity. Today, he works in a hospital under an interim contract.

**Figure 5:**
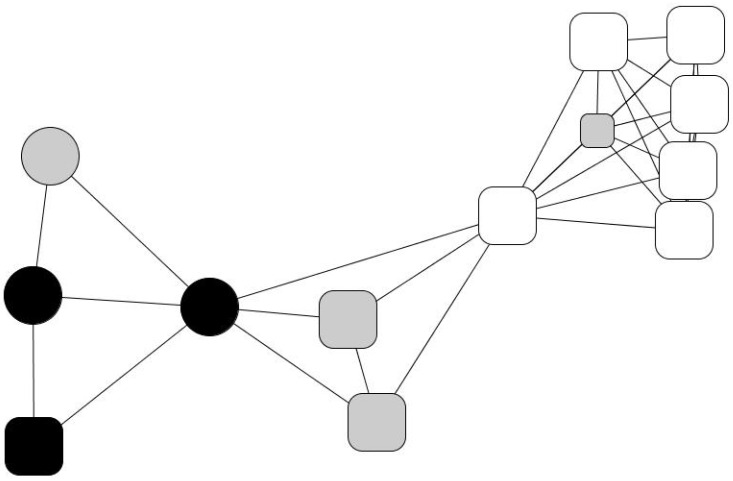
Andres’ personal network. Node color indicates type of relationship: family members (black), friends and acquaintances (gray), and professionals (white). Node size indicates the ego’s perception of alter’s status: big size (ego perceives that “alter has a better socioeconomic situation than me”), average size (ego perceives that “alter has a similar situation as me”) and small size (ego perceives that “alter has a worse socioeconomic situation than me”). Node shape indicates time of relationship: triangle (less than 1 year of relationship), square (between 1 and 5 years of relationship), rounded rectangle (more than 5 years) circle (lifetime).

Andrés’ network (Figure 5), similar to Julio’s, contains a large concentration of professionals (46%). Eulalia, a social worker, with the highest network centrality, mediates between the institution and his personal world (friends, a brother, and a few relatives). Three groups may be identified: one formed by friends (30%), another by social workers (47%), and one-third by relatives (14%). However, he considers he does not have close interactions with anyone apart from 7% of his acquaintances, whom he meets regularly. His network is slightly feminine (61% are women), and he perceives that 92% of his contacts enjoy better socioeconomic situations than him. However, 54% of his contacts (everyone except the professional staff) have barely any formal educational background (30% have no training, 23% have only basic training) nor a comfortable socioeconomic position. Thus, there is an evident socioeconomic divide between his institutional (right-hand side) and personal relationships (left-hand side).

However, social exclusion is not always related to addictions, as the cases of Evaristo and Joaquín show. They both depart from a socially disadvantaged situation, which may partly explain their difficulties in establishing long-term relationships. Alone, after long-term unemployment, they turned to charities.

**Figure 6:**
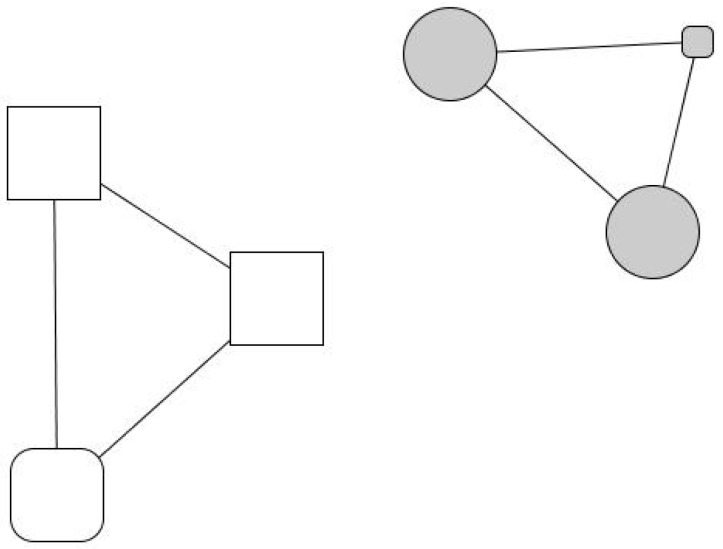
Evaristo’s personal network. Node color indicates type of relationship: family members (black), friends and acquaintances (gray), and professionals (white). Node size indicates ego’s perception of alter’s status: big size (ego perceives that “alter has a better socioeconomic situation than me”), average size (ego perceives that “alter has a similar situation as me”) and small size (ego perceives that “alter has a worse socioeconomic situation than me”). Node shape indicates length of relationship: triangle (less than 1 year of relationship), square (between 1 and 5 years of relationship), rounded rectangle (more than 5 years) and circle (lifetime).

Evaristo, a man in his fifties, had been married three times, had no children, and during his life had worked as a cook in several restaurants. However, he never established lasting labor nor social ties. After his last divorce, aimless, he suffered severe depression, lost his job, and ended up in a social shelter, where he started a relationship with Africa, another charity user whom he was planning to marry and start again from scratch, probably with a small restaurant—i.e., the same story that had been repeated so many times before in his case (see Figure 6).

Joaquín, a man in his sixties, had been raised in orphanages and, as a teenager, received training as a plasterer and bricklayer. He did not have a happy childhood, frequently being disobedient and suffering bad treatment and punishments. Since leaving the institution, he never managed to establish any stable social relationship. Aged sixty, alone, unemployed, and ill, he resorted to charities. His network has only six active contacts, three women and three men—two recent relationships, the rest being long-term acquaintances with whom he does not feel “very close”. He perceives most of his contacts as having a better socioeconomic situation than himself and, although he thinks these people would help him unconditionally, he recognizes that they “have their own problems”. In his network too, we observe two asymmetric social spheres: one made up of past friends with a similar or worse situation than himself and another made up of professionals, mainly women, with significantly better socioeconomic situations. His minimal personal network reflects an intense relational vulnerability.

### 3.4. Support Relations among Users, Volunteers, and Professionals

Here, we will summarize some of the interactional recurrences found in our sample. First, there is an evident deficiency of social bonds, both strong ties (family members, close friends, or stable partners) and weak ties (work relationships, neighborhoods, acquaintances). Often, the loss of a loved one, usually a parent, triggers a process of socioemotional decline, as in the cases of Andrés and Kike with their father, Julián with his mother, and Gabino with both parents. When his father died, Andrés explains, “I got into a labyrinth that I didn’t know how to get out of. The only exit was alcohol: you keep drinking and drinking and, in the end, you don’t remember anymore”.

Second, we observe a disproportionate presence of professionals in virtually all networks. These professionals provide care and support and have a crucial role in bridging the relationship between the user and his or her family, showing a commitment that often trespasses on the working day and job position: e.g., they visit their families, answer phone calls at any time, and take their charges to recreational activities such as the theatre. However, the blurring of the boundaries between work and leisure among professionals generates two opposite attitudes, both having reasonable rationales and justifications. On the one hand, some professionals affirm that establishing a strong bond is not a problem, while on the other hand, others believe they must encourage it to promote users’ social inclusion. For instance, one social worker, Roberto, wondered, “Why we don’t we talk and relate with them equally?” Yet others, however, prefer to maintain the boundary as a matter of professionalism. Francisco, the manager of a social shelter, put it this way: “Ethically, I cannot be a friend. It is something I must avoid to maintain an equal position in relation to everyone. Of course, this contradicts the guidelines that we try to give them: empathy, and putting ourselves in their place.” Similarly, Carmen, responsible for the addiction treatment center, said: “Our role is what it is. We have to avoid creating affective bonds that may confuse them”.

Third, gender plays a relevant role in diverse ways. Feminized networks (i.e., networks with a disproportionate number of female contacts) including MEH-affected males are widespread, suggesting a gender bias in the distribution of caregivers (professionals and volunteers) and aid depositaries (users). On the other hand, although severe exclusion often occurs among single adult males with a past of substance abuse and/or mental disorders (nine out of the twelve cases), less frequent female cases also show higher levels of violence and abuse. These findings are congruent with the opinion of mainstream research (see [20]).

Fourth, we noticed a significant absence: even though a large proportion of Humancare’s tasks rely on volunteering (generally retired women), we have not been able to document the presence of volunteers in any network. Rosa, a 59-year-old volunteer, thinks they should detach themselves emotionally from close interactions with users: “You have to try not to cross [the line]. Otherwise, you take them [the users] home”.

Fifth, although users tend to describe contexts of social care as competitive and insecure spaces where theft, rivalry, or arguments are not uncommon, social and collaborative ties of different types and intensities are also observed. Gregor accompanied another disabled user to solve some administrative problems; Enrique, a disabled homeless man, was cared for by many users at the social shelter; and users used to exchange tobacco, help, and information. Likewise, in long-term care contexts (shelters, social apartments, soup kitchens, etc.), we observed two types of dyadic ties. On the one hand, heterosexual romantic relationships play an essential role and provided mutual support and protection. During the fieldwork, we documented seven cases in our sample, including those of Inma and Gregor, Evaristo and Africa, and Sara and her indigent partner. These couples are often ephemeral because the circumstances are not suitable: they occur in transitional spaces and without privacy. On the other hand, dyadic relations of friendship are also observed, generally between adult males who share care spaces for a sustained period (e.g., Julio and Andrés).

Sixth, outside these protected niches, we find dependency relationships with children or elderly parents that imply high degrees of commitment and responsibility: examples include Inma or Sara and their children, or Gregor and his sick and elderly mother. Conversely, we also find ambivalent toxic links, emotionally loaded, particularly in cases of drug addiction and alcoholism. Julio wanted to get rid of an old friend whom he considered a bad influence. Gregor had a very conflictual relationship with his father, while Inma had one with his mother, and Monroy had one with his entire family. However, even though there was hardly any contact, these relationships were very present in their testimonies.

Finally, despite the traumatic contextual circumstances of the street, most narrate experiences in which they have received help from anonymous people. On one occasion, an old lady gave Eduardo a hot macaroni dish, and another man left him a €50 bill in the cardboard where he slept. Inma and Gregor encountered “good and altruistic people, who offered us alms and conversation (…) especially people with fewer resources: elderly women, teenagers, or poor people from the neighbourhood”. From time to time, Monroy, who is still living on the street, receives free second-hand books from the corner’s bookseller and food from strangers. Kike was supported by a philanthropist lawyer, and Eduardo considered that people, in general, “have treated me very well”.

## 4. Discussion

### 4.1. Sample

Most of the features highlighted by the mainstream literature are recognizable in our sample: primarily male, above forty, with some history of alcohol and/or drug abuse, with a high prevalence of physical and mental illness, a history of long-term unemployment, and variable amounts of time living on the streets [20,37]. Divorce, family breakdown, and early separation from the family are other risk factors that may trigger homelessness in our sample [19]. Cases involving females are less common, as expected, but these present a high prevalence of psychiatric morbidity, substance abuse, anxiety disorders, and being the victims of sexual and physical violence before or during the situation of homelessness [19] (p. 490). The low or non-existent educational level indicated by Muñoz and Vázquez [26] is also noticeable in our sample. However, we also found notable exceptions of individuals with medium-high educational backgrounds (Monroy, Kike, or Gabino), indicating more diversity than expected [10].

### 4.2. Measuring Relational Vulnerability through Personal Network Analysis

We anticipated that the networks of those affected by MEH would share similar characteristics with other matched groups of poor [13] (p. 465) in terms of size (i.e., their personal networks would be *smaller* than those who are not poor) [38], homophily (i.e., the individual’s contacts sharing similar socioeconomic situations and statuses) [14], and temporality [15]. All these characteristics are present in our cases as well.

Our sample suggests a relationship between the level of one’s social exclusion and the size of one’s personal network: the greater the degree of social exclusion, the smaller the network. In other words, extreme social exclusion manifests itself in the degree of relational dissolution [18] and the lack and weakness of social support [3]. Thus, the presence of strong ties (family and friends who usually provide essential resources), so fundamental as a safety net for the individual, is in some cases not only negligible but also severely limited in their ability to provide any support. Frequently, the individual departs from a vulnerable social situation of relative isolation (i.e., singleness, orphanhood, absence of children, early deaths of relatives, unstructured families, etc.) or belongs to a socioeconomically disadvantaged family with limited capability to provide material support [10]. Relationships with parents are rare, either because they have passed away or because the relationship itself has become deeply eroded. Siblings are not a robust factor in support either, whether because the relationship is conflictual or simply because they cannot help. In only two cases (Kike, Gabino) did we find a good relationship, highly reciprocal, with siblings (younger siblings, in fact), but only after overcoming an addiction. The high number of singles and the absence of children are also noteworthy, limiting both current and future support. However, at the time of the interview, some individuals were involved with new, probably ephemeral partners: during our fieldwork, we documented two couples among the respondents (Inma and Gregor, Evaristo and Africa) and three respondents with children from previous relationships (although they did not have custody anymore: Sara, Inma, and Gregor), while the rest, nine of the eleven interviewed, had neither children nor a known partner. Relationships with distant relatives are similar to those observed with other acquaintances: economic support is limited due to a lack of resources (i.e., homophilic contacts), although sometimes, distant relatives such as uncles or cousins might provide sporadic material support.

In these personal networks, we also observe relationships that accentuate their relational vulnerability. On the one hand, there are what we call key nexuses or unusually intense blood-related ties of attachment and dependence, whose disappearance can cause a devastating emotional impact. On the other hand, we observe ambivalent relationships that may put greater emotional stress on the individual, such as ties of dependence observed between ego and third parties, such as children, sick relatives, or the elderly, at the same time increasing the feeling of frustration because most of the needs generally cannot be addressed. Finally, we also observe “toxic links” [39] that constitute a form of negative social capital [40] because they subtract emotional or material resources and generate conflict and stress in close relationships: e.g., ambivalent love–hate relationships with relatives. Although these relationships are present in any network, their relational impact is potentially more significant among those affected by MEH.

Although isolation is not itself a sign of social exclusion, the personal networks in our cases are also limited in terms of resource provision (economic capital), degree of education (cultural capital), and heterogeneity of contacts. Both the perceived socioeconomic status (i.e., how the individual perceives their contacts’ socioeconomic situations) and alter’s educational level (i.e., their contacts’ education and professional training) indicate a considerable level of homophily (i.e., ego tends to relate to people of similar socioeconomic status). Homophily is not a specific characteristic of this vulnerable group, but in our cases, it plays a fundamental role as a cumulative factor in disadvantage [41].

### 4.3. Presence of Caring Professionals in the Personal Networks of People Experiencing MEH

In our sample, the disproportionate presence of caring professionals in their charges networks is striking, balancing the lack of socio-emotional support from strong ties. However, the professional often adopts the role of ‘fictitious kin’, forming a false family tie that emulates or replaces ordinary kinship roles, responsibilities, and care. Fictitious kinship is not uncommon in deprived sociocultural milieus such as *compadrazgo* in Mexico [42] or the familiar relationships depicted by Stack [43] among the Afro-American populations of marginal neighbourhoods, or the ascription in specific charity contexts of quasi-parental roles performed by volunteers on users, as in a soup kitchen or in maternal and child-care services [33]. However, in the context of MEH-related health care, the hybridity between strong (family) and weak (professional) ties reaches its greatest complexity.

### 4.4. Gender Bias

On the one hand, we detect a gender bias in the distribution of roles among caregivers (primarily women) and users (mostly male), showing that care giving is still a predominantly female occupation that sometimes takes on a maternal dimension. On the other hand, the caregiver role implies not only a risk “of blurring professional boundaries” [17] but of deep and unsolved contradictions for the practitioners in terms of commitment and ethics, balancing the promotion of existing social networks, such as partnerships or friendship against the high emotional costs of personal involvement. It is also very remarkable that in the networks we analyzed, no volunteers of the charity organization appear. This does not necessarily mean that volunteers do not play an essential role in users’ lives. It implies that users and volunteers do not establish lasting and close relationships of the sort we observe between professionals and users.

### 4.5. Incomplete Isolation

It is noteworthy that even among these severely excluded populations, total ostracism is rare. As Firdion and Marpsat point out, surveys and other research findings show that the “homeless do have social proximity to other people who are living in conditions of poverty and economic insecurity, confirming that the homeless cannot be considered as forming a distinct category” [10] (p. 583). Similarly, in our cases, sporadic contact with family members, although often conflictual, is common [44]. Furthermore, this contact is often recovered or reinforced in specific situations (in the event of an illness or the death of a family member, for example), as shown in the cases of Inma, Gregor, and Monroy. On the other hand, in contexts of exclusion, not all interactions are negative. Implicit norms of reciprocity, mutual aid [34], loyalty, and complicity [45] operate in these spaces, although the lack of material resources limits the scope of exchange. These collaborations function between users and often come from anonymous individuals in the form of donations and charitable actions. Thus, this kind of help remains a kind of hidden support on a small scale that does not always appear in the analysis of social networks but arises when a qualitative analysis is performed [35].

### 4.6. Challenges for Long-Term Care Services

For those affected by MEH, long-term care presents many and complex challenges. Probably the main challenge is the access to care services itself, which is often hampered by administrative problems (the lack of medical records, for instance) [16], structural violence [46,47,48], and the client’s distrust of institutions. The latter is sometimes caused by such violence but also by substance abuse and mental disorders [29].

There are also a few challenges regarding relational vulnerability. In addition to the high rate of suicide attempts, 100% of the interviewees suffered several physical and psychological ailments, from malnutrition, anemia, HIV, cancer, disability, sclerosis, or fibromyalgia to compulsive disorders, manic depression, and suicidal tendencies. There not only exists a relationship between homelessness and mental disorders [49], but we have also observed that the more time the homeless individual spends on the streets, the greater the degree of his or her decline in health, well-being, and basic skills [28] (p. 122). Thus, creating social bonds is often considered a secondary priority after meeting urgent needs in terms of health, shelter, and food. However, as we have observed, those affected by MEH suffer a wide range of problems: addictions, family breakdown, or mental illness, among others [50]. As some researchers suggest, this kind of care involves not a sequence of phases but a parallel process in which it is also vital to establish social anchors because social care and the creation of new connections prevent the aggravation of psychological distress and substance abuse [51].

This type of intense care requires efficient networking between professionals, volunteers, and institutions (parishes, public and private services, etc.), which is a process of “accompaniment” through a long and slow circuit through which the individual (if successful) must gradually recover skills, routines, and self-confidence in other contexts that may be challenging, such as social shelters, health treatments and detoxification, shared apartments, work groups, or therapy. In extreme cases, users require permanent hospitalization and a demanding level of dedication in terms of professional care. Here, we found a twofold challenge. One is the creation of successful bridges of social inclusion beyond the institution. As we have seen, long-term spaces of care (e.g., soup kitchens or social shelters) usually provide protected niches from the rest of modern and competitive society and offer an opportunity to socialize and establish relationships without the emotional impact of long-term and emotionally dense relationships [30]. Nevertheless, establishing social relationships with the broader society is problematic for several reasons, such as stigma, users’ financial deprivation, or the increasing individualism of society, particularly when people are unemployed. The second challenge is that in a neoliberal context of public cutbacks aimed at reducing costs [52] and faced with the resurgence of far-right political movements, investments in the underprivileged are treated with suspicion.

## 5. Conclusions

As Anderson suggests, in considering homelessness as one more step in the process of decline that is exclusion, the relational appraisal allows a sharper reflection on “seriously problematic life events and care needs and associated support” (2001: 2, quoted in [19] (p. 644). The present paper contributes to the knowledge of a group on which little relational data are available by carrying out an interactive analysis of the ways in which long-term care transforms these networks. We have described the specific personal network particularities of those affected by MEH in terms of their size (relatively small), structure (with fewer social components than in ordinary networks, but with a disproportionate presence of professionals), and support (scarce and conditioned by cumulative disadvantage and specific bonds). Our data strongly support the idea that those affected by MEH require both preventive and restorative social interventions [22] instead of palliative and reproductive measures [53]. Although charity and the altruistic acts of strangers perform a fundamental task in this regard, the real challenge is to achieve social inclusion on a basis of equality that is guaranteed by a public health system.

Our synchronic social network analysis only provides a static picture of the experience of homelessness, which is a dynamic and long-term process. In this respect, more data and a longitudinal approach are required to observe significant transformations at the relational level so as to understand better how these transformations in their turn are connected with individual well-being. We finish by requesting Official Statistical institutions systematic collection of reliable data from the segments of the population affected by MEH to devise appropriate mitigation policies.

## Data Availability

All data are available at Nebula secure server: https://nebula.uab.cat/share/, accessed on 24 September 2021.
